# Determinants of antiretroviral treatment adherence among HIV/AIDS patients: a multisite study

**DOI:** 10.3402/gha.v6i0.19570

**Published:** 2013-03-15

**Authors:** Bach Xuan Tran, Long Thanh Nguyen, Nga Hoang Nguyen, Quynh Van Hoang, Jongnam Hwang

**Affiliations:** 1 Institute for Preventive Medicine and Public Health, Hanoi Medical University, Hanoi, Vietnam; 2 Authority of HIV/AIDS Control, Ministry of Health, Hanoi, Vietnam; 3 Hanoi School of Public Health, Hanoi, Vietnam; 4 School of Public Health, University of Alberta, Edmonton, AB, Canada

**Keywords:** HIV/AIDS, adherence, antiretroviral treatment, drug users, adherence aid, Vietnam

## Abstract

**Introduction:**

Adherence to antiretroviral treatment (ART) is vital in achieving virological treatment success. This study assessed the prevalence of optimal ART adherence and its determinants among HIV/AIDS patients in Vietnam.

**Method:**

A cross-sectional survey was conducted with 1,016 HIV/AIDS patients at seven hospitals and health centers providing antiretroviral treatment services in three provinces, including Hanoi, Hai Phong, and Ho Chi Minh City. Self-reported medication adherence was measured using a 30-day visual analog scale (VAS) and 7-day missed-doses questions.

**Results:**

The mean adherence VAS-score was 94.5 out of 100 (SD=8.2), ranging from 40 to 100%. The rate of suboptimal adherence was 25.9%. The rate of missed-doses was 25.2%. In multivariate analysis, increased perceived self-efficacy, use of mobile phone alarms, and reminders from family members were associated with optimal adherence; higher CD4 level, single status, and unstable employment were associated with suboptimal adherence.

**Conclusion:**

High rate of suboptimal adherence observed in this study highlights the importance of adherence support interventions during ART. The use of mobile phone reminders, involvement of relatives, and HIV self-management training programs have the potential to improve ART adherence in Vietnam.

Globally, rapid expansion and early access to antiretroviral treatment (ART) services have resulted in a dramatic decrease in HIV-related mortality and mobility (1, 2). However, the current regimens are life-long requirement of strict compliance by patients to achieve treatment success and prevent drug resistance. In resource-scarce settings, where second and third line ART regimens and viral load monitoring are limited, routine assessment of and interventions for patients’ adherence have become one of the biggest priorities in delivering ART services.

Vietnam has a growing HIV epidemic, which emerged initially in drug using populations (3, 4). By 2011, there were approximately 198,000 HIV cases reported, and 46,000 patients in need of treatment received free-of-charge medications at ART clinics (5). ART service delivery has been decentralized, and ensuring its quality and efficiency is critical (6). A recent study in two districts found that only 75% of patients reported optimal adherence, defined by 95% of prescribed medication (7). In the literature, factors associated with ART adherence have been identified, which include: substance use, depression, medication side-effects, stigma and discrimination, and the lack of family and social supports (8–11). These structural and social barriers varied across settings; thus, it is necessary to characterize contextual factors affecting ART adherence if one would like to design an effective intervention. To date, there is little information available on adherence to ART across health service administration levels in Vietnam. Using data from the 2012 Vietnam HIV Services Users Survey, we assessed adherence to ART, and determinants of suboptimal adherence in three metropolitan areas of Vietnam.

**Policy implications**

1. More than a quarter of HIV/AIDS patients reported suboptimal adherence to antiretroviral treatment and that suggests supporting interventions should be maintained throughout the course therapy.
2. Simple adherence measures, self-reported VAS and missed doses, can be used for periodically assessing ART adherence in clinical practice in Vietnam.
3. Enhancing patients’ self-efficacy, involving family members, and using mobile phone alarms are potential strategies for supporting ART adherence.

## Methods

### Study settings

We used data from the 2012 HIV Services Users Survey (HSUS), which was administered in three HIV provinces of Vietnam, including Hanoi, Hai Phong, and Ho Chi Minh City (12, 13). The HSUS aims to evaluate various dimensions of HIV services quality and outcomes from the perspective of patients that informs HIV program management and policy development in Vietnam. The most HIV cases were found in Ho Chi Minh City, which had more than 41,000 HIV cases and 23.1% of newly detected HIV cases nationally in 2009. Hanoi, which is the capital city of Vietnam and has a population of about 6 million, had 23,000 HIV cases. Hai Phong, a port city of about 1.8 million citizens on the Red River Delta in northern Vietnam, contains a large drug using population of approximately 9,500, among whom approximately 50% were HIV-positive (14, 15).

### Study design and participant recruitment

A cross-sectional study was conducted in seven hospitals and health centers, which were selected based on the following criteria: 1) they were central-, provincial- or district-level hospitals or health centers; 2) they have been providing ART services, and 3) a sufficient number of HIV/AIDS patients attend each clinic. One central hospital (National Hospital of Tropical Diseases), three provincial hospitals (Dong Da, Viet Tiep, and Ho Chi Minh Hospital for Tropical Diseases), and three district health centers (Tu Liem, Binh Tan, and Le Chan District Health Centres) were selected. All HIV-positive inpatients and outpatients who were registering for care or taking ART at selected hospitals were eligible for the study. The ART clinics are integrated into general health care facilities; therefore, if patients with HIV/AIDS have other health problems than AIDS-related ones (opportunistic infections, ART side effects, or HIV counseling), they will be admitted to corresponding departments. Thus, inpatients at ART clinics mostly came with infectious infections, and usually stayed for short periods, and accounted for a small number in our sample (16). We selected patients who were present at the clinics during the study period until reaching at least 100 patients per site and 200 patients per clinic at the national level. A total of 1,016 volunteer participants were interviewed face-to-face by well-trained interviewers using a structured questionnaire. The interview took approximately 30–40 min. Among 1,016 study participants, 60% of them were from Hanoi, while 20% were from Hai Phong and Ho Chi Minh City, respectively. To control the quality of data, we conducted periodic and ad-hoc supervision during data collection. Data were managed using a computerized program using double-check entering and automated codes.

### Measures and instrument

The questionnaire included three main parts. The first part included questions about patient socio-economic characteristics (age, gender, marital status, religion, education, income, and employment) and HIV-related characteristics (HIV/AIDS stages, history of drug use, the duration of knowing their HIV status, and ART access and duration). The second part assessed patients’ adherence using two measures. First, patients reported their adherence over the past 30 days on a 100-point visual analog scale (VAS), where 0 indicated complete non-adherence and 100 indicated perfect adherence (17). The threshold for optimal adherence was defined at 95% and above (16). Second, we assessed the dose-adherence by asking patients two simple questions: if they missed taking any pills, or if they took pills 2 hours later than scheduled over the past 7 days. Dose adherence was considered optimal if patients reported ‘no’ to both questions, and suboptimal if they answered ‘yes’ to any question.

The third part included questions about medication adherence self-efficacy, reasons for missing doses, and adherence aids. Adherence self-efficacy was assessed using a global rating question: ‘Over the past month, how confident have you been that you can comply with ART?’ which included a five-level response option from poor to excellent. Potential aids were also explored to examine which might be feasible and effective measures for enhancing ART adherence.

### Statistical analysis

We examined the prevalence of ART adherence and suboptimal adherence, socio-demographics, and HIV-related characteristics of respondents in descriptive analysis. *Multivariate linear and logistic regression* was used to determine factors associated with VAS score and suboptimal adherence. Dependent variables of interest included: (a) VAS score, (b) having VAS scores last month less than 95%, and (c) reporting missed doses within the last 7 days (16). We identified an ‘a priori’ set of candidate variables, including socioeconomic status, health status and ART, self-efficacy, and adherence supporting measures. We applied a stepwise forward model building strategy where variables were selected based on the log-likelihood ratio test, and we adopted a *p*-value <0.1, and excluded variables at *p*-values >0.2 (18). Those variables remained in the reduced model, but those that were not statistically significant at *p*<0.05 have still been presented if they imply clinical- or policy- importance.

### Ethical considerations

This project is a part of the ‘Cost and cost-effectiveness of HIV/AIDS care and treatment policy options in Vietnam’ research project. The use of these data was approved by the Authority of HIV/AIDS Control, Ministry of Health, Vietnam.

## Results

### Characteristics of participants

Of the 1,016 patients interviewed, 36.2% were female, and the average age was 35.4 (SD=7.0). One-third of patients were married or living with partners (34%); most (86.5%) had at least secondary education. More than half of respondents were self-employed or working without a basic salary; 15.6% were officers, white-collar employees, or workers; and more than 22.6% were farmers or unemployed (Table 1) .

**Table 1:** Characteristics of participants

Characteristics	Mean	SD
Age	35.4	7.0
Years living with HIV (SD)	5.7	3.7
Household's income per capita^a^ (thousand Vietnam Dong)	2,042.4	53.8
	*N*	%
Education		
Below high school	556	54.7
High school	307	30.2
Vocational training, college, university	153	15.1
Marital Status		
Single	142	14.0
Have partners	325	32.0
Married	325	32.0
Widow/divorced/separated	224	22.1
Employment		
Unemployed	181	17.8
Freelancer^b^	534	52.6
Officers, white collars, workers	158	15.6
Farmers	49	4.8
Others	94	9.3
Religion		
Non	768	75.6
Buddhism	206	20.3
Others	42	4.2
Clinical characteristics		
History of opiate drug abuse	468	46.1
Current opiate drug abuse	325	86.0
HIV stages		
Asymptomatic	126	12.4
Symptomatic	508	50.0
AIDS	382	37.6
Taking ART	902	88.8
CD4 count		
≤200	249	31.1
200<CD4≤350	249	31.1
350<CD4≤500	194	24.2
>500	109	13.6

a 1 USD=20,500 Vietnam Dong (2011).

b Self-employed, non-salary basis.

With regard to clinical characteristics, 46.1% of respondents reported a history of drug abuse. Over one third of respondents had advanced to AIDS stage (37.6%), half were in the symptomatic HIV stage (50%), and 12.4% were asymptomatic. The mean duration since first HIV-positive diagnosis was 5.7 years (SD=3.7 years). Most respondents (902/1,016; 88.8%) were taking ART at the time of survey. The mean duration on ART was 3.03 years (SD=2.1 years). Over one third of participants (33.5%) had been enrolled in an ART program for less than 24 months. The percentage of patients with CD4 counts less than 200 cells/ml was quite high (31%), and less than 350 cells/ml was 62.17% (Table 1).

### ART adherence, barriers, and adherence support strategies

Assessment of ART therapy adherence is summarized in Table 2. The mean percentage of adherence of 30-day VAS score was 94.5 (SD=8.2), with a range of 40.0–100.0. Using a score of 95% to determine optimal adherence, 74.1% patients reported optimal adherence in the month prior to the survey. With regard to missed doses, 8.5% of patients reported forgetting pills in the last 7 days, and 16.7% reported taking pills late.

**Table 2:** Summary statistics for self-reported ART adherence

Measures		
1. VAS Adherence Score (*N*=876)		
Mean	94.5	
SD	8.2	
Min	40.0	
Max	100.0	
Proportion of different VAS adherence Levels	*N*	%
<75%	35	4.0
75–84%	44	5.0
85–94%	148	16.9
≥95%	649	74.1
2. Missed doses last 7 days	*N*	%
Forget doses (*N*=857)	73	8.5
Take medicines late than 2 hours (*N*=850)	142	16.7

Table 3 presents various reasons for non-adherence or missed doses among HIV/AIDS patients. The two most common reasons were being busy with work (33%), and forgetting to take medicine (21.5%). Other commonly cited reasons included not taking pills along with them (14.5%), sleeping through dose (11.5%), and travelling (6.5%). Most of respondents used some strategy to remind them to take their medication. The main devices used for adherence supports were mobile phone's alarms (62.2%), followed by an alarm clock (22.7%). Furthermore, 25.4% patients were reminded by relatives to take their medication, 1.5% had friends or peers remind them, and 25.4% relied only upon themselves to take their medication.

**Table 3:** Reasons for non-adherence to ART and patients’ adherence support strategies

	*N*	%
Reasons for non-adherence (*N*=200)		
Busy working	66	33.0
Forgot	43	21.5
Pills not with patients	29	14.5
Slept through dose	23	11.5
Travelling	13	6.5
Medications make me sick	6	3.0
Not alarmed because mobile phone turned	6	3.0
Did not want others to see me take pills	5	2.5
Drunken	4	2.0
Ran out of medications	3	1.5
Hospital admission	1	0.5
Take care of children	1	0.5
Strategies for supporting adherence (*N*=919)		
Set the mobile phone alarm	572	62.2
Reminded by relatives	233	25.4
Self-help	233	25.4
Set the alarm clock	209	22.7
Friends, peer-supporters come to remind	14	1.5
Other	5	0.5
None	4	0.4

### Factors associated with non-adherence to ART among HIV/AIDS patients

Table 4 presents the associations of individual characteristics with non-adherence to ART in multivariate analyses. Socioeconomic status was significantly associated with missed doses over the last 7 days. A higher likelihood of missed doses was found among patients who were single, self-employed, and in the poor group (vs. the poorest). As for clinical characteristics, we found that patients with a CD4 count of between 350 and 500 cells/ml had significantly lower odds of non-adherence compared to those with a CD4 count of less than 200 cells/ml. Patients who had a CD4 count>500 cells/ml had a slightly lower VAS score than the 350–500 cells/ml group.

**Table 4:** Factors associated with ART non-adherence among HIV/AIDS patients

	VAS Score		Non-adherence last month		Missed dose last week	
			
	Coefficient	95% CI	OR	95% CI	OR	95% CI
Health system level						
Provincial vs. acentral					1.52	(0.94; 2.47)
Income per capita (aQ1-poorest)						
Poor	−1.22	(−2.51; 0.06)	1.56	(0.90; 2.68)	1.85	(1.04; 3.28)*
Richest			0.55	(0.30; 1.02)		
Marital status (asingle)						
Married	0.74	(−0.36;1.83)			0.44	(0.25; 0.78)**
Divorced, separated	1.3	(−0.46; 3.05)			0.37	(0.15; 0.90)*
Widow(er)					0.37	(0.16; 0.87)*
Employment (aunemployment)						
Freelancer					1.86	(1.06; 3.25)*
Officers	1.74	(−0.26; 3.74)				
Workers					1.96	(0.81; 4.75)
Others	1.81	(0.09; 3.53)*	0.5	(0.20; 1.28)	2.06	(0.83; 5.13)
Religion: Buddhism vs. ano religion	1.1	(−0.19; 2.39)	0.49	(0.26; 0.89)*	0.42	(0.22; 0.81)**
Drug abuse: no vs. yes					0.68	(0.42; 1.11)
CD4 cells count (a<200 cells/mL)						
350 → 500			0.49	(0.27; 0.88)*		
>500	−1.71	(−3.16; −0.26)*	1.52	(0.82; 2.84)		
HIV stage: symptomatic vs. aasymptomatic					1.54	(0.94; 2.52)
Years taking ART			1.1	(0.97; 1.25)		
Self-efficacy for adherence (apoor)						
Moderate	6.37	(0.88; 11.86)*				
Good	17.1	(12.32; 21.87)**	0.24	(0.09; 0.64)**	0.17	(0.06; 0.47)**
Very good	20.8	(16.06;25.55)**	0.03	(0.01; 0.10)**	0.09	(0.03; 0.24)**
Excellent	23.01	(18.23; 27.79)**	0.03	(0.01; 0.08)**	0.02	(0.01; 0.07)**
Adherence supporting measures						
Alarm clock: yes vs. ano					2.68	(1.54; 4.66)**
Mobile phone: yes vs. ano	0.89	(0.02; 1.99)*				
Reminded by family members: yes vs. ano					0.44	(0.25; 0.80)**

a Reference group.

* Significant at 5%;

** significant at 1%.

We found that patient's higher levels of self-efficacy for ART adherence were associated with higher VAS scores, and were inversely associated with non-adherence within the last month and missed doses within the last week. Additionally, the use of reminder strategies, such as mobile phone alarms and reminders from family members, was associated with optimal adherence. Mobile phone use could improve the VAS score although the role of chance cannot be ruled out. Using an alarm clock was associated with a higher risk of having missed doses.

## Discussion

This study showed a high prevalence of suboptimal adherence to ART among HIV/AIDS in three provinces of Vietnam. There was a combination of various barriers to ART adherence, including working, not bringing pills along, sleeping through the dose, and travelling. We observed that type of employment, marital status, religion, income, and immunological status were significantly associated with non-adherence. Additionally, self-efficacy, familial supports, and the use of mobile phones are potential strategies for enhancing ART adherence.

Our findings were consistent with a previous study from two districts in northern Vietnam, which reported 24.9% non-adherence and 29.0% not-on-time-adherence (7). Our results are also in line with adherence assessments in other settings in Asia and Africa (11, 19–21). We identified a number of individual factors significantly associated with non-adherence and missed doses during ART. Notably, lack of employment is a common contributor to ART non-adherence in Vietnam (22). In addition, cultural barriers, stigma and discrimination of people at work places, were associated with reduced ART adherence (22).

The strong association we observed between self-efficacy and ART adherence observed in this study suggests that interventions related to patient self-efficacy may prove efficient. Furthermore, we observed a slightly poorer adherence among patients with higher CD4 counts (>500 cells/ml). This could be explained by the hypothesis that the patient was careless when they realized that their health status recovered. Therefore, regular assessment and supporting interventions to promote ART adherence should be maintained throughout the course therapy. One important finding from this study is the identification of effective strategies used to adhere to ART regimens. Particularly, the majority of patients used mobile phones as a reminder (62.2%), and this was associated with a higher level of ART adherence. Mobile phones are used widely throughout Vietnam and have become an integral device that is almost never left at home (23). Thus, an automated alarm on a mobile phone could help remind patients, regardless of setting. Many patients were self-employed (52.6%) and are highly mobile; thus, mobile phones could prove a feasible and effective tool for adherence support. Besides mobile phones, family members should also be involved in ART treatment support for HIV/AIDS patients, as they can help provide medication reminders for daily treatment, support to overcome side-effects, and help promote a stable life.

The strengths of this study include a large number of patients sampled from central, provincial, and district ART clinics in three metropolitan areas of Vietnam. However, there were several limitations that should also be acknowledged. First, ART adherence was self-reported, which was susceptible to recall and social desirability biases. In the literature, self-reported adherence tended to produce estimates of adherence that were 10–20% higher than those from electronic drug monitoring (24, 25). To minimize these biases, we employed interviewers who were not affiliated with the selected clinics, and explained clearly to the patients the purposes of the study prior to interviewing them. In addition, we used a high cut-off for adherence level (≥95%) and a 1-month time period to measure the rate, which has proved effective to detect non-adherence (16, 25). Also, the visual analog scale had been previously validated in the Vietnamese context and showed convergent validity with the Adult AIDS Clinical Trials Group instrument (7). However, more refined measures of ART adherence such as pill counts and biomarker measurements may still be useful for further study. Further study assessing barriers to ART adherence may consider broader factors, such as clinical settings (connection with healthcare providers) and social settings (stigma, discrimination), or deeper personal factors (psychological characteristics). As for drug use behaviors, although this relied upon self-report, previous work has shown a high correlation between self-report and other measures, such as heroin-confirmation urine tests, in these settings (26). Finally, this convenient sample included few inpatients and did not include patients who had not accessed health facilities during the study periods, and who were in the rural areas, that limit the generalizability of study findings. Those inpatients attending ART clinics were mostly for opportunistic infection treatment over short periods of time that showed indifferent levels of adherence in comparison with outpatients. In practice, HIV information is confidential; thus, constructing a sample frame of HIV/AIDS patients for recruiting a representative sample is not possible. Because of this, findings of this study could reflect only the current situation in these selected clinics.

In conclusion, the prevalence of suboptimal adherence was high. Simple adherence measures, self-reported VAS and missed doses, can be used for periodically assessing ART adherence in clinical practice in the Vietnamese settings. Interventions to improve ART adherence should be implemented, especially in a large drug using population. Familial supports and mobile phone reminders hold potential in supporting adherence to ART.
